# To use or not to use? Understanding doctoral students’ acceptance of ChatGPT in writing through technology acceptance model

**DOI:** 10.3389/fpsyg.2023.1259531

**Published:** 2023-10-26

**Authors:** Min Zou, Liang Huang

**Affiliations:** ^1^School of Foreign Languages, Beijing Institute of Technology, Beijing, China; ^2^Department of Public Administration, Southeast University, Nanjing, China

**Keywords:** ChatGPT, writing, technology acceptance model, artificial intelligence-based chatbot, doctoral students

## Abstract

While artificial intelligence-based chatbots have demonstrated great potential for writing, little is known about whether and how doctoral students accept the use of ChatGPT in writing. Framed with Technology Acceptance Model, this study investigated doctoral students’ acceptance toward ChatGPT in writing and the factors that influence it. The questionnaire survey revealed a high intention to use ChatGPT in writing among doctoral students in China. The findings further indicated that attitude was a significant predictor of behavioural intention to use ChatGPT in writing and mediated the impacts of perceived usefulness and perceived ease of use on it. Perceived ease of ChatGPT use was in turn influenced by students’ past ChatGPT use experience. This study provides powerful evidence for the applicability of Technology Acceptance Model in the acceptance of ChatGPT in writing. The results have significant implications for leveraging ChatGPT for writing in higher education.

## 1. Introduction

Artificial intelligence (AI) technologies play a crucially important role in the increasingly digitalized world (Lee et al., 2022; Farrokhnia et al., 2023). As a generative AI chatbot, ChatGPT is a large language model that can autonomously learn from data and produce human-like texts (van Dis et al., 2023). It can converse on a wide range of topics and generate human-like responses after training huge quantities of text data (OpenAI, 2023). Ever since its release in November 2022, ChatGPT has sparked debates about its implications for education (Farrokhnia et al., 2023; Tlili et al., 2023; van Dis et al., 2023). While ChatGPT can potentially transform educational practices by providing a baseline knowledge of diverse topics (Tlili et al., 2023) and facilitating personalized, complex learning (Farrokhnia et al., 2023), it may supply incorrect texts, encourage cheating, and threaten academic integrity (Dwivedi et al., 2023; van Dis et al., 2023). The controversies have made ChatGPT “the most high-profile and controversial form of AI to hit education so far” (Williamson et al., 2023, p. 2).

Writing has been one of the most influenced domains in the ChatGPT era (Taecharungroj, 2023; Yan, 2023). While writing plays an important role in higher education (Kirkpatrick, 2019), it has been oftentimes considered challenging for language learners, especially for those who learn and use English as an additional language (Ma, 2021). Prior research has suggested that chatbots are effective in addressing this challenge, since they could supply meaningful guidance and substantive feedback to support language learners to write at their own pace in a less anxiety-inducing environment and improve writing quality (Guo et al., 2022; Zhang et al., 2023). As a chatbot powered by generative AI, ChatGPT has demonstrated improved abilities than earlier chatbots (e.g., ELIZA) to understand natural language, generate appropriate responses, and engage in free-flowing conversations throughout the writing process, hence opening a new avenue for writing practice (Barrot, 2023; Su et al., 2023). As succinctly summarized by Imran and Almusharraf (2023), ChatGPT is “a complete package from generation to final proofreading and editing of writing material” (p.2). Nevertheless, till now, scarce attention has been paid to the acceptance and usage of ChatGPT in English writing—a daunting but critical work facing doctoral students (Kirkpatrick, 2019). Little is known about whether and how doctoral students intend to use ChatGPT in writing and the key determined factors. Informed by Technology Acceptance Model (TAM; Davis, 1989), the present study seeks to fill the void by addressing the following two questions: (1) how is the doctoral students’ acceptance intention to ChatGPT in writing? (2) what factors may influence doctoral students’ acceptance intention to ChatGPT in writing? Such information is important, as the individuals’ intention to adopt and use AI technology is critical to improving teaching and learning of writing (Cheng, 2019; Yan, 2023).

## 2. Literature review

### 2.1. The use of ChatGPT in writing

Chatbots, computer programs or AI systems designed to simulate human conversations and interact with users via natural language, have gained considerable attention and increasingly applied in writing in the past decade (Zhang et al., 2023). Chatbots have demonstrated great potential as a writing assistant and learning partner in writing classrooms, as they can provide a broad array of language choices and feedback to students’ writing process and make students feel less stressed about their writing performance in the learning process (Guo et al., 2022). ChatGPT was developed in 2022 as a novel chatbot rooted in Generative Pre-training Transformer architecture, and outperforms early chatbots in terms of the capability for understanding and producing human-like texts as well as providing feedback on long texts (Dwivedi et al., 2023; Farrokhnia et al., 2023; Su et al., 2023; Tlili et al., 2023). Such affordances make it a powerful writing assistant and writing tool (Barrot, 2023; Dergaa et al., 2023; Imran and Almusharraf, 2023). As shown in Taecharungroj’s (2023) analysis of early reactions on Twitter, ChatGPT has been most frequently used for writing, such as essays and articles.

Given the close link between ChatGPT and writing, a growing body of research has been undertaken to investigate the benefits and threats associated with the use of ChatGPT in writing. Piloting ChatGPT for academic writing, Bishop’s (2023) user experience demonstrated that ChatGPT is effective in explaining well-known concepts, translating between languages, giving timely and personalized feedback, adjusting the style and tone of texts to imitate different writers, and perfecting the mechanics of writing, thereby enhancing writing efficiency and promoting writing quality. Zooming into the use of ChatGPT in second language writing context, Barrot (2023) and Su et al. (2023) further unpacked the potential of collaborating with ChatGPT in writing classrooms. For them, ChatGPT has taken into consideration various writing constructs, such as pragmatics, coherence and syntax, and could support the structural, dialogical and linguistic aspects of quality writing by assisting students in topic generation, outline preparation, content revision, proofreading and post-writing reflection. Taking stock of the research on ChatGPT in academia, Dergaa et al. (2023) and Imran and Almusharraf (2023) highlights the need to leverage ChatGPT as a valuable writing assistant tool to support the writing process and enhance academic writing.

Notwithstanding the benefits, the use of ChatGPT in writing has also raised concern for inaccurate and unintelligent responses, academic integrity, learning loss and educational inequality (Dwivedi et al., 2023; Farrokhnia et al., 2023; Tlili et al., 2023). As noted by the developer itself (OpenAI, 2023), “ChatGPT sometimes writes plausible-sounding but incorrect or nonsensical answers.” Such incorrect and biased information can mislead students and be further incorporated into their writing, thereby harming knowledge practice and science progress (Tlili et al., 2023; van Dis et al., 2023). Another limitation of using ChatGPT in writing is associated with its unintelligent responses, typified by its frequent use of irrelevant statements, template rigidity of writing, and insufficiencies in emotional depth in writing (Barrot, 2023). Also, ChatGPT does not always reference sources appropriately and cannot be held accountable for their work, which raises pertinent issues concerning plagiarism and academic integrity (Dergaa et al., 2023; van Dis et al., 2023; Williamson et al., 2023; Yan, 2023). Additionally, the generative nature of ChatGPT allows students to complete writing assignments simply through unwitting copy-and-paste, and hence results in learning loss, especially when students become too reliant on the AI-powered chatbot for convenience (Barrot, 2023). Likewise, using ChatGPT in writing could lead to educational inequality (Dwivedi et al., 2023). Focusing on ChatGPT’s text generation functionality, for example, Yan’s (2023) research showed the undergraduates were much concerned with its impact on educational equity, given that writing teachers may not effectively distinguish texts produced by students from those produced by ChatGPT.

While the above user cases and scholarly discussions are helpful in unpacking the potentials and pitfalls of using ChatGPT in writing, the research into ChatGPT is still at its early stage (Barrot, 2023). Little empirical research has been conducted to examine the socio-technical aspects of using ChatGPT in writing. Since writing is essential to doctoral education (e.g., Kirkpatrick, 2019) and subject to the advances in AI technologies (Yan, 2023), it is necessary to explore and examine doctoral students’ intention toward ChatGPT and the influencing factors. Such information could shed light on doctoral students’ acceptance of ChatGPT in writing, and generate useful insights to leverage ChatGPT and other similar generative AI technologies for the teaching and learning of writing in higher education.

### 2.2. Technology acceptance model

User acceptance refers to the prospective users’ predisposition toward using technology (Lee and Lehto, 2013). TAM, emerging from the theory of reasoned action, has become an influential socio-technical model that seeks to identify and explain the end-users’ acceptance of technology (e.g., Cheng, 2019; Granić and Marangunić, 2019). In TAM, individuals’ acceptance of a particular technology is operationalized as their behavioural intentions to use it (Lee and Lehto, 2013). TAM postulates that people’s actual usage of technology is determined by their behavioural intentions. Behavioural intentions, in turn, are jointly determined by people’s attitudes and perceived usefulness (Davis et al., 1989). Attitude towards technology underscores individuals’ affective reactions to and evaluation of the use of the technology (Ajzen, 1991; Lee and Lehto, 2013) and it is closely related to one’s intrinsic motivation (Davis et al., 1992). If people have a more favourable attitude toward the technology, they are more likely to form positive intentions to use it (Davis et al., 1989; Estriegana et al., 2019). Perceived usefulness is people’s belief about the extent to which using the technology will improve their performance (Davis, 1989). It is a type of extrinsic motivation in determining technology acceptance and technology usage behaviour (Davis, 1989; Lee and Lehto, 2013). That is, if students believe that using the technology will improve their performance in writing, they tend to have a positive inclination to use it. The perceived usefulness is also hypothesized to have a positive influence on attitudes and thus affect behavioural intentions (Davis et al., 1989). If the technology is viewed as useful in enhancing writing performance, students are apt to appraise the technological means positively and inclined to use it (Estriegana et al., 2019). Therefore, this study proposes the following hypotheses.

*Hypothesis 1*: Attitude towards using ChatGPT in writing would significantly and positively influence students’ behavioural intention to use ChatGPT in writing.

*Hypothesis 2*: Perceived usefulness of using ChatGPT would significantly and positively influence students’ behavioural intention to use ChatGPT in writing.

*Hypothesis 3*: Perceived usefulness of using ChatGPT would significantly and positively influence students’ attitude towards using ChatGPT in writing.

*Hypothesis 4*: Attitude towards using GPT would significantly mediate the effects of perceived usefulness on students’ intention to use ChatGPT in writing.

Furthermore, TAM posits that attitude is jointly determined by perceived usefulness and perceived ease of use which refers to “the degree to which a person believes that using a particular system would be free of effort” (Davis, 1989, p.320). In TAM, perceived ease of use is assumed to have a significant effect on perceived usefulness and attitudes, resulting in increased behavioural intention (Davis et al., 1989; Alfadda and Mahdi, 2021). If the technological tool is perceived to be easy to use, students tend to consider it helpful and develop a favourable attitude, thereby demonstrating a strong inclination to use it in writing (Alfadda and Mahdi, 2021). Subsequently, the following hypotheses can be proposed.

*Hypothesis 5*: Perceived ease of use would significantly and positively influence students’ perceived usefulness of ChatGPT in writing.

*Hypothesis 6*: Perceived ease of use would significantly and positively influence students’ attitude towards using ChatGPT in writing.

*Hypothesis 7*: Attitude towards using GPT would significantly mediate the effects of perceived ease of use on students’ intention to use ChatGPT in writing.

Meanwhile, a number of studies have revealed a strong and direct association between perceived ease of use and behavioural intention (Granić and Marangunić, 2019). In Yang and Wang’s (2019) study, for instance, the perceived ease of use showed a significant and positive impact on students’ behavioural intention to use machine translation. As argued by Shiau and Chau (2016), when people perceive that using a technological tool does not require much effort, they will be more intended to use it. Hence, the following hypothesis is proposed.

*Hypothesis 8*: Perceived ease of use would significantly and positively influence students’ behavioural intention to use ChatGPT in writing.

According to Davis et al. (1989), perceived usefulness and perceived ease of use are influenced by a range of external variables, among which experience is one best studied external factor (Abdullah and Ward, 2016). The existing literature suggests that experience influences both learners’ perceived usefulness (e.g., Chang et al., 2017; Yang and Wang, 2019) and perceived ease of use of educational technologies (e.g., Purnomo and Lee, 2013). For instance, Chang et al. (2017) found that students who have more experience in using computers tend to demonstrate more positive perceptions regarding the ease of use and usefulness of e-learning. Hence, this study assumes that students who have experience in using generative AI chatbots are more prone to understand usefulness of ChatGPT and become more proficient in using it in EFL writing. The following hypotheses are accordingly proposed.

*Hypothesis 9*: Past ChatGPT use experience would significantly and positively influence perceived usefulness of ChatGPT in writing.

*Hypothesis 10*: Past ChatGPT use experience would significantly and positively influence perceived ease of using ChatGPT in writing.

**Figure 1:**
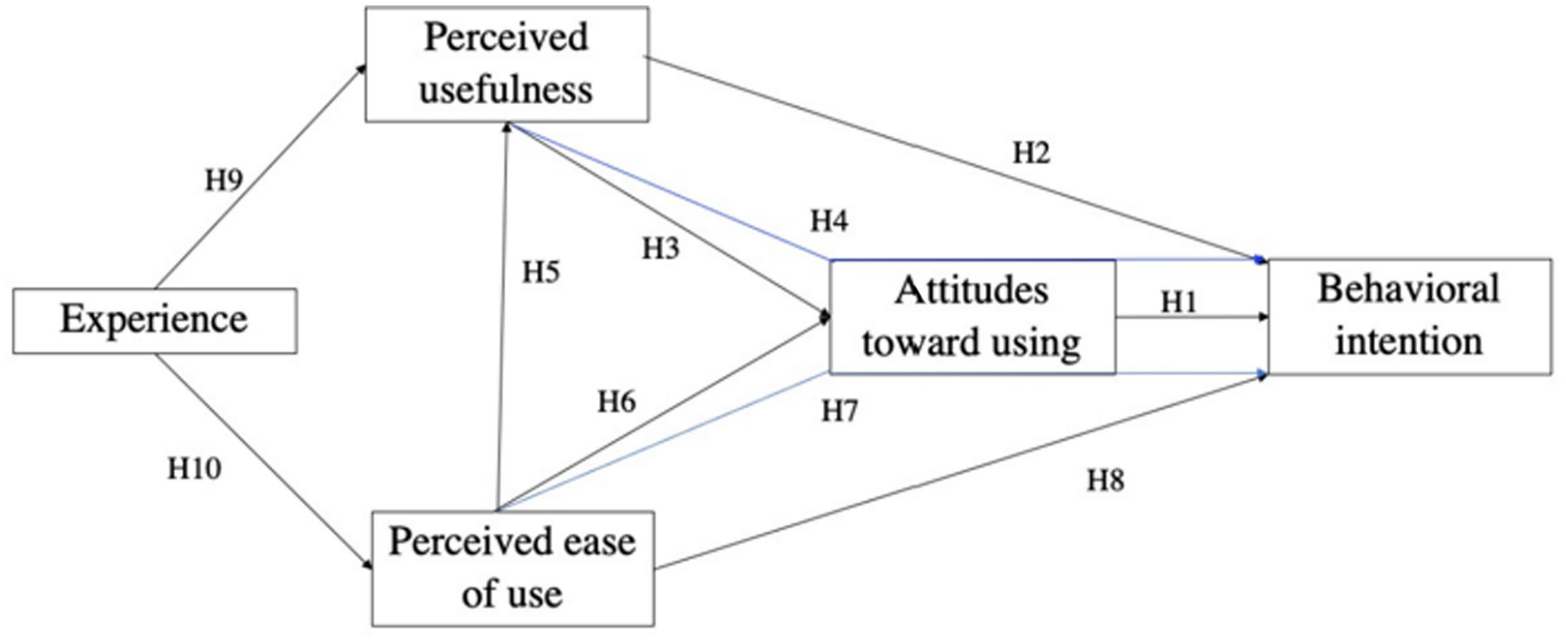
Conceptual model.

Taken together, and in line with the existing literature on TAM, a conceptual model is formulated in the present study (see Figure 1).

## 3. Research methodology

### 3.1. Participants

A total number of 242 doctoral students (151 males and 91 females) participated in the study through convenience samplings in one technological university in China. The students, ranging from 24 to 43 in age, were enrolled in the compulsory course entitled Writing for Academic Success taught by the first author. The course aims to empower doctoral students to improve English for academic writing skills. The participants were from different disciplinary backgrounds, such as computer science, mechanical engineering, materials science, economics, and education.

### 3.2. Measures

To determine doctoral students’ acceptance of ChatGPT in writing and the factors influencing it, an online survey was administered in March 2023. The survey instrument consisted of two sections subsuming questions pertaining to demographic profiles (gender, major, and past ChatGPT use experience) and those concerning the constructs in TAM. The survey items in the second part were adapted from Davis (1989), Edmunds et al. (2012), Lee and Lehto (2013), and Rafique et al. (2020), and in light of the usage of ChatGPT in writing. In the second section, the respondents indicated their agreement level on every item by recording their response in a 6-point Likert scale, ranging from “1” (Strongly Disagree) to “6” (Strongly Agree).

#### 3.2.1. Perceived ease of ChatGPT use in writing

Perceived ease of ChatGPT use in writing was measured based on a five-item scale adapted from Davis (1989). The five items (e.g., “I think ChatGPT is easy to use”) showed high reliability (Cronbach’s α = 0.854). In light of Hu and Bentler’s (1999) study, the Confirmatory Factor Analysis (CFA) results suggested good construct validity (*χ*^2^ = 9.445, df = 5, RMSEA = 0.061, CFI = 0.991, TLI = 0.982), with factor loading ranging from 0.608 to 0.821.

#### 3.2.2. Perceived usefulness of ChatGPT in writing

Perceived usefulness of using ChatGPT in writing was assessed by a five-item scale adapted from Davis (1989) and Rafique et al. (2020). The five items (e.g., “Using ChatGPT would enable me to finish English writing assignments effectively”) demonstrated high reliability (Cronbach’s α = 0.841). The CFA results showed good construct validity (*χ*^2^ = 4.254, df = 5, RMSEA = 0.000, CFI = 1.000, TLI =1.000), with factor loading ranging from 0.637 to 0.785.

#### 3.2.3. Attitude towards using ChatGPT in writing

Attitude towards using ChatGPT in writing was measured on a five-item scale adapted from Edmunds et al. (2012). The five items (e.g., ‘I like using ChatGPT while writing in English’) demonstrated excellent reliability (Cronbach’s α = 0.915). As indicated by Hu and Bentler (1999), the CFA results showed good construct validity (*χ*^2^ = 10.184, df = 5, RMSEA = 0.065, CFI = 0.994, TLI = 0.987), with factor loading ranging from 0.775 to 0.879.

#### 3.2.4. Behavioural intention to use ChatGPT in writing

Behavioural intention to use ChatGPT in writing was measured on a five-item scale adapted from Lee and Lehto (2013) and Rafique et al. (2020). The five items (e.g., “I intend to use ChatGPT to improve my English writing ability in the future”) showed high reliability (Cronbach’s α = 0.871). According to Hu and Bentler (1999), the CFA results demonstrated good construct validity (*χ*^2^ = 7.976, df = 5, RMSEA = 0.050, CFI = 0.995, TLI = 0.990), with factor loading ranging from 0.659 to 0.838.

#### 3.2.5. Past ChatGPT use experience

In the present study, students’ past ChatGPT use experience was operationalized as whether the students had used ChatGPT *de facto* at the time of data collection. It was measured via one item, i.e., “Have you ever used ChatGPT before?” The respondents indicated their past experience on a yes-no scale (Yes = 1, No = 0).

### 3.3. Data analysis

SPSS 24.0 and Mplus 7.4 Software were used for data analysis. First, the SPSS software was used to conduct descriptive analysis and correlation analysis. Then, the Mplus software was utilized to construct structural equation modelling (SEM), with a view to calculating relationships among focus variables and conduct mediation analysis. For mediation analysis, bias-corrected bootstrapping method with 2000 times of resampling was employed to calculate the point estimates of the confidence intervals regarding the mediating effects. In light of Hu and Bentler’s (1999) research, the fit of the model was evaluated by the following cut-off values: Root mean-square error of approximation (RMSEA) < 0.08; Tucker-Lewis index (TLI) > 0.90; and comparative fit index (CFI) > 0.90.

Additionally, Harman’s single factor test was conducted by SPSS software to exclude possible common variance bias. The results showed that less than 50% (46.80%) of the total variance of variables were explained after all the items were loaded into one factor, indicating no need to control common variance bias (Mat Roni, 2014).

## 4. Results

### 4.1. Preliminary analysis

The descriptive statistics of all variables are presented in Table 1. Except for past ChatGPT use experience, the other four focus variables’ score fall between 3.954 and 4.159, indicating mid-to-high levels on behavioural intentions, attitudes, perceived usefulness and perceived ease of use regarding ChatGPT. Particularly, the students reported the highest score on behavioural intention (M = 4.159), revealing doctoral students’ high intention to use ChatGPT in writing in this study.

**Table 1:** Results of descriptive statistics and correlation analysis.

	1	2	3	4	5
1. Behavioural intention to use ChatGPT in writing	1				
2. Attitude towards using ChatGPT in writing	0.784^***^	1			
3. Perceived usefulness of ChatGPT in writing	0.632^***^	0.701^***^	1		
4. Perceived ease of ChatGPT use in writing	0.590^***^	0.688^***^	0.660^***^	1	
5. Past ChatGPT use experience	0.093	0.132^*^	0.032	0.163^*^	1
Mean	4.159	3.954	4.106	4.017	0.463
SD	0.917	0.953	0.930	0.820	0.500

Standardized coefficients are reported.

* *p* < 0.05

*** *p* < 0.001.

As suggested by the correlation matrix in Table 1, perceived ease of ChatGPT use (γ = 0.590, *p* < 0.001), perceived usefulness of ChatGPT (γ = 0.632, *p* < 0.001), and attitude towards using ChatGPT (γ = 0.784, *p* < 0.001) were significantly and positively correlated with students’ behavioural intention to use ChatGPT in writing. Besides, both perceived ease of ChatGPT use (γ = 0.688, p < 0.001) and perceived usefulness of ChatGPT (γ = 0.701, *p* < 0.001) were significantly and positively correlated with doctoral students’ attitude towards using ChatGPT in writing. Perceived ease of ChatGPT use was significantly and positively correlated with perceived usefulness of ChatGPT in writing (γ = 0.660, *p* < 0.001). Moreover, past ChatGPT use experience was significantly and positively correlated with students’ perceived ease of ChatGPT use (γ = 0.163, *p* < 0.05), but it was not significantly correlated with perceived usefulness of ChatGPT in writing (γ = 0.032, *p* > 0.05).

### 4.2. Structural equation modelling

**Figure 2:**
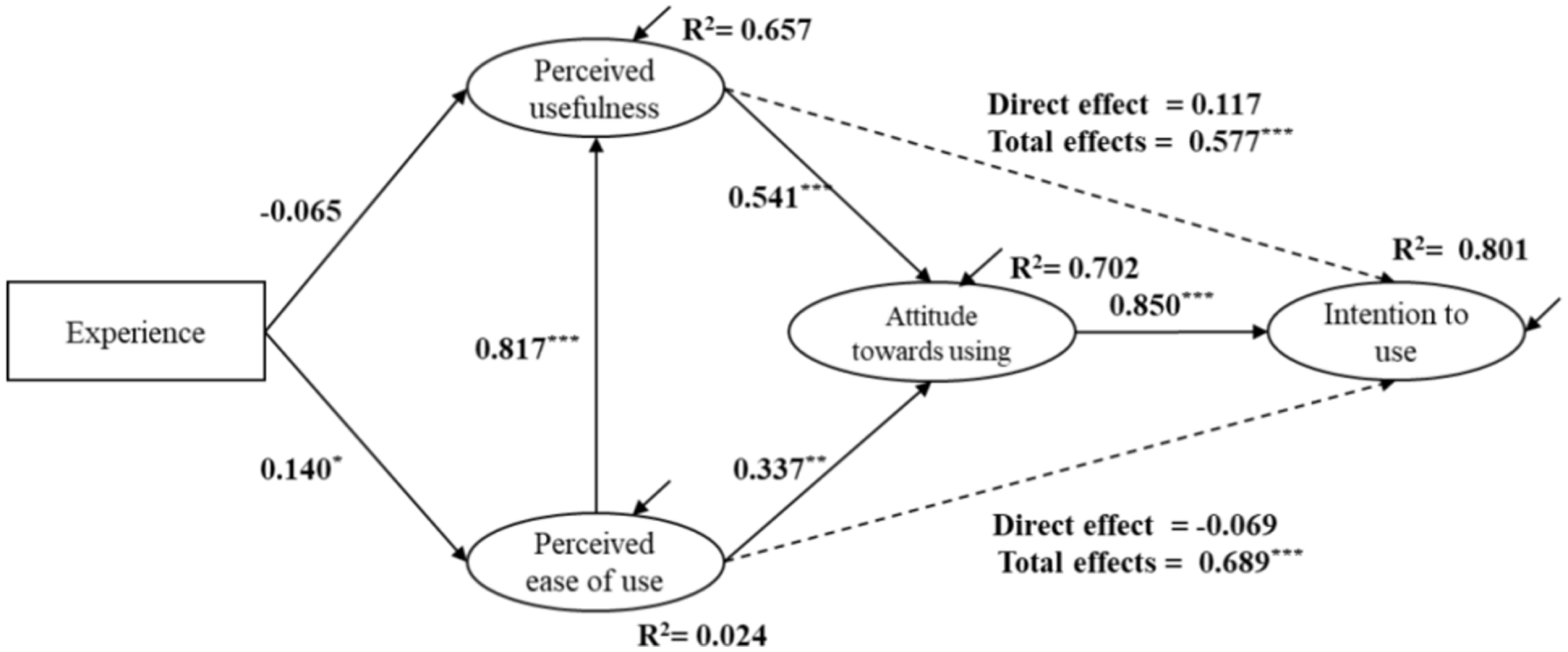
Modified model for behavioural intentions to use ChatGPT in writing. Standardized coefficients are reported. *p < 0.05, **p < 0.01, ***p < 0.001.

SEM analysis was conducted to examine the relationships among focus variables with gender being controlled for all the structural relationships. As shown in Figure 2, the model had a high explanation for variance in students’ behavioural intention to use ChatGPT in writing (80.1%), attitude towards using ChatGPT (70.2%), and perceived usefulness of ChatGPT (65.7%), respectively, and a low explanation for variance in perceived ease of ChatGPT use (2.4%). The model fit indices (χ^2^ = 350.545, df = 198, RMSEA = 0.056, CFI = 0.951, TLI = 0.943) indicates a good SEM model fit.

Perceived attitude towards using ChatGPT in writing had significant and positive impacts on students’ behavioural intention to use ChatGPT in writing (β = 0.850, *p* < 0.001), supporting H1. Perceived usefulness of using ChatGPT had significant total influences on students’ behavioural intention to use ChatGPT (β = 0.577, *p* < 0.001), but did not have significant and direct influences on it (β = 0.117, *p* > 0.05), thus rejecting H2. However, perceived usefulness of ChatGPT had positive and significant influences on students’ attitude towards using ChatGPT in writing (β = 0.541, *p* < 0.001), thus supporting H3. Besides, perceived ease of use had significant and positive effects on students’ perceived usefulness of ChatGPT in writing (β = 0.817, *p* < 0.001), thus supporting H5. Perceived ease of ChatGPT use had positive and significant influences on students’ attitude towards using ChatGPT in writing (β = 0.337, *p* < 0.001), thereby supporting H6. Perceived ease of use had significant total influences on students’ behavioural intention to use ChatGPT (β = 0.689, *p* < 0.001) but had no significant and direct influence on it (β = −0.069, *p* > 0.05), rejecting H8. In addition, past ChatGPT use experience had significant and positive influences on students’ perceived ease of using ChatGPT in writing (β = 140, *p* < 0.05) but had no significant influence on perceived usefulness of ChatGPT (β = −0.065, *p* > 0.05). Therefore, the results supported H10 but rejected H9.

Additionally, results of mediation analysis (Table 2) show that students’ attitude towards using ChatGPT significantly mediated the effects of perceived usefulness of ChatGPT on their behavioural intention to use ChatGPT in writing (β = 0.460, *p* < 0.001, 95% CIs: 0.149 to 0.771), hence supporting H4. It also significantly mediated the influences of perceived ease of ChatGPT use on students’ behavioural intention to use ChatGPT in writing (β = 0.287, *p* < 0.05, 95% CIs: 0.022 to 0.552). Thus, H7 was supported.

**Table 2:** Results of mediation analysis.

	β	S.E.	95% Confidence intervals
Perceived usefulness → behavioural intention to use ChatGPT in writing (Direct effect)	0.117	0.179	[−0.233, 0.467]
Perceived usefulness → attitude towards using → behavioural intention to use ChatGPT in writing	0.460^**^	0.159	[0.149,0.771]
Perceived ease of use → behavioural intention to use ChatGPT in writing (Direct effect)	−0.069	0.137	[−0.337, 0.200]
Perceived ease of use → attitude towards using → behavioural intention to use ChatGPT in writing	0.287^*^	0.135	[0.022, 0.552]

Standardized coefficients are reported.

* *p* < 0.05

** *p* < 0.01.

## 5. Discussion

While ChatGPT has ignited debates about its applications in education (e.g., Farrokhnia et al., 2023), it remains unknown whether students are willing to use it or not in writing. This research contributes to the existing literature by investigating Chinese doctoral students’ acceptance toward ChatGPT in writing and its major influencing factors. Through the lens of TAM, the present study revealed a strong intention to use ChatGPT in writing among doctoral students, which was affected by their attitudes, perceived usefulness, and perceived ease of use. The findings provide a deeper understanding of doctoral students’ acceptance inclination toward ChatGPT and other generative AI chatbots in writing in higher education.

Although ChatGPT remains new, the doctoral students demonstrated a strong intention to use it in writing. This corroborates Taecharungroj’s (2023) finding that ChatGPT has been mainly used in the writing domain. Students’ high behavioural intentions might be attributed to the affordances of ChatGPT for writing. As shown in prior research (e.g., Bishop, 2023; Yan, 2023), ChatGPT could help students to brainstorm ideas, obtain timely and personalized feedback, translate language items, and improve written drafts. This makes it a potential mediation tool for doctoral students to write more fluently and effectively in the publish-or-perish system (Kirkpatrick, 2019).

Consistent with our prediction, doctoral students’ attitude towards using ChatGPT in writing was found to be a significant predictor of behavioural intention. While a number of prior studies have removed attitudes from TAM due to its weak role in mediating the effects of perceived usefulness and perceived ease of use on behavioural intention (e.g., Lee and Lehto, 2013; Yang and Wang, 2019), this study found that attitude not only directly influences behavioural intention but also mediates the impacts of perceived usefulness and perceived ease of use on it. The finding lends support to the original TAM (Davis et al., 1989). It also supports Ajzen’s (1991) argument that personal attitude towards a behaviour functions as a major determinant of people’s intentions to perform it. In other words, when doctoral students have more positive evaluation of using ChatGPT in writing, they are more willing to perform the behaviour. Also, as suggested by the expectancy-value model of attitudes (Ajzen, 1991; Ajzen and Fishbein, 2008), people’s attitude is further determined by salient beliefs regarding the outcome of performing the behaviour and attributes associated with the behaviour, such as the cost and effort incurred by performing it. In this sense, positively valued outcomes and easier management of the technology could strengthen users’ affective reactions towards the technology and boost their sense of efficacy, hence contributing to their favourable attitude towards it and the resultant increasing behavioural intention (Davis et al., 1989). As shown in this study, doctoral students’ attitude towards using ChatGPT in writing, shaped by the perceived usefulness and ease of use, played an important role in mediating their effects on students’ intention to use ChatGPT in writing.

Furthermore, the results revealed that perceived usefulness and perceived ease of use had significant total influences on students’ behavioural intention to use ChatGPT in writing. This echoes the central role of perceived usefulness and perceived ease of use in the adoption process of technology in prior research examining TAM (Cheng, 2019; Granić and Marangunić, 2019; Alfadda and Mahdi, 2021). Nevertheless, the study found no significant direct influence of them on doctoral students’ behavioural intention. Instead, they only influenced behavioural intention through attitudes. This surprising finding is inconsistent with previous studies on people’ acceptance of educational technology (e.g., Estriegana et al., 2019; Yang and Wang, 2019). This might be due to the fact that some researchers (Davis, 1989; Lee and Lehto, 2013; Chang et al., 2017; Yang and Wang, 2019) did not include the attitude variable in their models and consequently failed to explore its mediating effects. Another plausible explanation might be that ChatGPT remains new, and early adopters use ChatGPT mainly because it facilitates inherently enjoyable and interesting experience (Taecharungroj, 2023; Tlili et al., 2023). In other words, the use of ChatGPT at this stage is primarily intrinsically motivated (Davis et al., 1992). Accordingly, the expected outcome of using ChatGPT for enhancing writing performance at the extrinsic level and perceived ease of using ChatGPT at the technical level could be instrumental, when such beliefs catalyse intrinsic motivations and when using ChatGPT in writing appeals to individuals (Ryan and Deci, 2000).

Also, the study found that perceived ease of use was found to be significantly and positively influenced perceived usefulness of ChatGPT in writing. This is analogous to Rafique et al.’s (2020) study, in which users’ perceived ease of using mobile library applications had a significant influence on perceived usefulness. By the same token, users’ perceived ease of using ChatGPT in writing could greatly shape the perceived usefulness (Davis et al., 1989). If doctoral students consider it challenging to apply ChatGPT in writing, they are likely to hold that ChatGPT has little effect on their writing. When they perceive ChatGPT easy to use, they tend to regard it as useful and helpful for writing.

In addition, this study extends prior research on TAM by including experience as an external factor to enhance the model explanatory power. Doctoral students’ past ChatGPT experience is proved to be a significant predictor for perceived ease of use. The more experienced the students are, the more positive they are about the ease of using ChatGPT in EFL writing. This is compatible with Purnomo and Lee’s (2013) study, where prior computer experience had a positive influence on learners’ perceived ease of use an e-learning system and such influence was stronger than that on perceived usefulness. The findings also support of argument Nelson’s (1990) that the acceptance of technology relies upon not only the technology itself but also individuals’ expertise in using it. Students with experience in using generative AI chatbots could employ the knowledge and skills obtained from prior experience to writing, develop a better personal control, and accordingly perceive it easier to use it in writing (e.g., Purnomo and Lee, 2013; Chang et al., 2017).

## 6. Conclusion

Despite the increasing interest in ChatGPT in educational settings, research on its acceptance is still scarce in education. Based on TAM, descriptive statistics, correlation analysis, and SEM were employed to gauge doctoral students’ acceptance of ChatGPT in writing and explore the influencing factors. Data analysis revealed a high-level intention to use ChatGPT in writing, shaped by doctoral students’ attitudes, perceived usefulness, and perceived ease of use. The present study could contribute to ChatGPT research in both theoretical and practical ways. Theoretically, the inclusion of experience in TAM helps to reveal the variables that could influence doctoral students’ adoption of ChatGPT in EFL writing. As our model explained 80.1% of the variance in behavioural intention, this study overall supports and advances the applicability of TAM in ChatGPT, a new technology in writing education.

Practically, the results of the study could also generate useful implications for technology developers, policy-makers, writing teachers, and doctoral students to leverage ChatGPT for the teaching and learning of writing. Doctoral students’ strong intention to use ChatGPT in writing suggest that ChatGPT may augment its function as an educational tool for writing in higher education. Considering the significant and strong effect of attitude on students’ behavioural intentions to use ChatGPT in writing, it is of necessity for educational institutions, writing teachers, and technology developers to be aware of students’ attitudes and increase their positive evaluation of and affective reactions towards using ChatGPT in writing. For instance, technology developer can make the usage of ChatGPT more innovative, enjoyable and interesting so as to create more positive attitudes and boost learners’ intrinsic motivation to use ChatGPT in writing. Given the increasing concerns for information, ethical and learning risks associated with ChatGPT (e.g., Barrot, 2023; Dwivedi et al., 2023) and doctoral students’ strong intention to use ChatGPT for writing, measures must be taken to mitigate such negative impacts of ChatGPT on doctoral students. For example, technology developers can strengthen the quality control of generated responses. Similarly, writing teachers need to provide trainings on effective, ethical and responsible use of ChatGPT in writing. Besides, perceived ease of use and perceived usefulness are found to have a significant influence on students’ attitude, which could further exert an effect on students’ intentions to use ChatGPT in writing. The sequential and circular influential relationship among the variables implies a need for technology developers to increase the usefulness and ease of using ChatGPT in writing to make it more functional and user-friendly. For example, technology developers can keep simplifying and optimizing the operation of ChatGPT based on user feedback and provide comprehensible instructions or use cases regarding how to apply ChatGPT to write more effectively and ethically. Instead of prohibiting the use of ChatGPT in writing, policy makers need to take into consideration the students’ voice and align their educational needs with the AI tool (EDUCAUSE, 2023). For writing teachers and institutional administrators, efforts to integrate ChatGPT in writing courses or training programs are needed to capitalize on ChatGPT’s affordances for writing and improve students’ ability to use ChatGPT as an effective writing assistant tool. Given the significant effect of past ChatGPT experience on perceived ease of ease, instructing doctoral students to increase their use of ChatGPT, and reflect upon and communicate the skills for utilizing ChatGPT to promote writing performance could be an effective way to develop their expertise in ChatGPT. Also, doctoral students can experiment with ChatGPT in a conscious manner, and record their hands-on experience to continuously improve the capability for effective and ethical use of ChatGPT for writing.

Regardless of the contributions, there are several limitations that need to be taken into consideration in future research. Firstly, while the study revealed a high intention to use ChatGPT in writing among doctoral students, it was exploratory in nature and only used questionnaires to gauge students’ acceptance of ChatGPT. Future research can thus employ case study research deign or mixed study research design and collect multiple sources of data (e.g., semi-structured interviews, user reflections, and screenshots) to obtain an idiosyncratic and in-depth understanding of students’ actual process and outcome of using ChatGPT in writing. Secondly, the present study was based on a sample of doctoral students from a science and technology university in China. The types of writing assignments they face and their needs for using ChatGPT to improve writing could be very different from other learner groups like undergraduates (Yan, 2023) and students in other countries, which limits the generalizability of this study. Therefore, future research can expand the sample scope to include students with varied educational levels and backgrounds to increase the generalizability and representativeness. It may also be interesting to conduct cross-section research to examine whether the level of use acceptance across different learner groups in the future. Thirdly, our data was collected from participants who interacted with ChatGPT shortly after the release of ChatGPT and who used ChatGPT primarily for its inherently enjoyable and interesting experience (Taecharungroj, 2023; Tlili et al., 2023). Given the increasing ethical, learning and information concerns concerning the use of ChatGPT in writing in academia (Barrot, 2023; Su et al., 2023) and students’ growing experience, knowledge and skills regarding ChatGPT, their attitudes, perceptions and intentions of using ChatGPT in writing may alter over time. Longitudinal research can be conducted to trace the development of knowledge concerning the use of ChatGPT for writing among doctoral students, and how such knowledge influences their attitudes towards, as well as perceptions and intentions of using ChatGPT in writing. Considering the doctoral students’ high intention to use ChatGPT for writing and the increasing concerns for information, ethical and learning risks associated with ChatGPT (e.g., Barrot, 2023; Dwivedi et al., 2023), it is also promising to explore effective ways to integrate ChatGPT in writing instruction and construct writing models to empower students to collaborate with ChatGPT in an effective, ethical and responsible manner.

## Data availability statement

The raw data supporting the conclusions of this article will be made available by the authors, without undue reservation.

## Ethics statement

The studies involving humans were approved by the ethics committee of the School of Foreign Languages, Beijing Institute of Technology. The studies were conducted in accordance with the local legislation and institutional requirements. The participants provided their written informed consent to participate in this study.

## Author contributions

MZ: Conceptualization, Funding acquisition, Investigation, Project administration, Writing – original draft. LH: Conceptualization, Formal analysis, Methodology, Software, Writing – review & editing.
